# Dual HER2 blockade with pertuzumab (P) and trastuzumab (T) in patients with HER2-positive metastatic breast cancer (mBC) relapsing after adjuvant treatment with T: results from a German non-interventional study (NIS) HELENA (NCT01777958)

**DOI:** 10.1007/s10549-022-06710-4

**Published:** 2022-09-12

**Authors:** Marc Thill, Pauline Wimberger, Andrea Grafe, Peter Klare, Kerstin Luedtke-Heckenkamp, Dietmar Reichert, Matthias Zaiss, Katja Ziegler-Löhr, Tanja Eckl, Andreas Schneeweiss

**Affiliations:** 1 Department of Gynecology and Gynecological Oncology, Agaplesion Markus Hospital, Frankfurt A. M, Germany; 2 Department of Gynecology and Obstetrics, TU Dresden and National Center for Tumor Diseases (NCT/UCC), Dresden, Germany; 3 German Cancer Research Center (DKFZ), Heidelberg, Germany; 4 Faculty of Medicine and University Hospital Carl Gustav Carus, Technische Universität Dresden, Dresden, Germany; 5 Helmholtz-Zentrum Dresden - Rossendorf (HZDR), Dresden, Germany; 6 Medical Center Nordhausen gGmbH, Nordhausen, Germany; 7 Gynecological Out-Patient Clinic, Berlin, Germany; 8 Center for Oncology and Hematology, Niels-Stensen-Clinics, Franziskus-Hospital Harderberg, Georgsmarienhütte, Germany; 9 Center for Oncology, Westerstede, Germany; 10 Practice for Interdisciplinary Oncology & Hematology, Freiburg, Germany; 11 Practice for Gynecological Oncology, Cologne, Germany; 12 Roche Pharma AG, Grenzach-Wyhlen, Germany; 13 National Center for Tumor Diseases (NCT), Heidelberg University Hospital and German Cancer Research Center, Heidelberg, Germany

**Keywords:** HER2-positive, Pertuzumab, Trastuzumab, Dual HER2 blockade, Metastatic breast, Cancer

## Abstract

**Purpose:**

NIS HELENA documented outcomes in clinical routine practice of first-line therapy with P plus T and docetaxel (D) of patients with advanced HER2-positive BC and prior (neo)adjuvant T.

**Methods:**

Between 06/2013 through 07/2016, 126 patients (in-label use of P at study start = full analysis set, FAS) in 81 German study sites were included. Intense documentation period was limited to 28 treatment cycles. Maximum follow-up (FU) was 24 months (mos). Safety was assessed in the safety set (SAF = eligible patients with at least one dose of P, *n* = 132). Median progression-free survival (PFS) was the main parameter of interest.

**Results:**

Mean age of FAS patients was 55.1 [30.7–80.2] years, 81.7% (95.2%) were < 65 (75) years of age. 51.6% of the FAS patients were hormone receptor-positive (HR+), 91.3% had distant, 73.0% visceral, and 18.3% non-visceral metastases. Median disease-free interval was 40.2 [6.6–95.9] mos. Effectiveness (FAS): Median PFS was 18.8 [15.1; 24.2] mos. Overall response rate was 64.3% (55.6; 72.1). Median overall survival was 55.9 mos [41.2, not reached]. Safety (SAF): 93.9% of patients had an adverse event (AE), 32.6% a serious AE (SAE). AEs related to P occurred in 53.8% of SAF, SAEs related to P in 13.6%. Diarrhea was the most frequently reported related (S)AE. There were 8 (6.1%) patients with a fatal AE.

**Conclusion:**

Based on the outcomes from NIS HELENA, results of dual blockade with P+T in patients relapsing after (neo)adjuvant T as reported from the CLEOPATRA study (NCT01777958) can be transferred to routine clinical practice. No new safety signals were detected.

## Introduction

Female breast cancer (BC) represents the most common neoplastic disease in the global population, with 2,261,419 new cases and 684,996 deaths worldwide in 2020 [1]. About every fourth newly diagnosed malignancy in women is BC [1]. Survival rates among patients differ based on molecular subtype and stage [2]. Amplification or overexpression of HER2 is observed in about 20% of invasive breast cancers [3] and is associated with a distinctly poor prognosis. The prognostic disadvantage relates to a more aggressive tumor biology, a higher risk of recurrence, a higher frequency of brain metastases and a shorter overall survival compared to other BC subtypes [4–6].

The discovery of the *HER2* oncogene represents one of the most important advances in BC research and is the foundation of HER2-directed therapies [7, 8]. Currently, several HER2-directed therapies such as the HER2-directed antibodies trastuzumab (T) and pertuzumab (P), tyrosine kinase inhibitors, and antibody–drug conjugates are available for the treatment of patients with HER2-positive (HER2+) metastatic BC [4]. Due to their complementary modes of action the combination of P and T is more effective compared to the single agents [9, 10].

Treatment guidelines and definitions of standards of care are based on results from randomized controlled studies. However, these are characterized by specific patient selection criteria and accurately defined assessment methods and intervals. Thus, data from clinical studies may not mirror effectiveness in routine clinical practice. The randomized controlled phase III trial (RCT) CLEOPATRA investigated the combination of P plus T and Docetaxel (D) versus T plus D plus placebo in patients with advanced HER2+ BC. Median progression-free survival (PFS) as assessed by an Institutional Review Facility (IRF) was 12.4 months (mos) in the control arm and 18.5 mos for dual HER2 blockade with P plus T [11]. A post hoc analysis reported a median PFS of 16.9 mos for patients with prior (neo)adjuvant T when treated with P plus T and D in first-line [12]. Dual HER2 blockade with P and T plus a taxane is the current first-line standard of care for patients with HER2+ mBC [6, 13, 14], including patients relapsing after (neo)adjuvant therapy [15].

The current non-interventional study (NIS) was designed to document effectiveness and safety of first-line dual HER2 blockade plus chemotherapy in routine clinical practice (hospital or outpatient setting) in patients with advanced HER2+ BC relapsing after prior (neo)adjuvant T, a patient group at a principally higher risk of disease progression. Median PFS was the main parameter of interest. Overall results are displayed descriptively including results from pre-defined subgroups. Patient-reported quality of life (QoL, Fact-B-Questionnaire) and decision criteria for choice of therapy by treating physician, according to pre-populated categories on the electronic case record form (eCRF) were also captured. The choice of first-line dual HER2 blockade was a decision taken by the participating physicians as part of their clinical routine. Course of treatment followed clinical routine according to the respective summaries of product characteristics.

## Materials and methods

This study was performed in line with the principles of the Declaration of Helsinki. Approval was granted by the Ethics Committee of the Chamber of Physicians of Hessen. Informed consent was obtained from all individual participants included in the study. 156 patients were enregistered into this NIS for screening from a total of 81 outpatient and hospital-based centers. For recruitment, all patients were screened for relapse of HER2+ BC presenting either with inoperable local recurrence or metastatic disease after prior T in the (neo)adjuvant therapy setting.

Primary objective of this NIS was to assess the median PFS of patients with advanced HER2+ BC relapsing after prior (neo)adjuvant T with adequate accuracy (point estimate ± 3 months). PFS was defined as the period between diagnosis of advanced HER2-positive breast cancer leading to initiation of P+T+Chemotherapy, and progression or death due to any cause. Reference basis was the median PFS of 16.9 mos, reported in the respective subgroup of patients in the CLEOPATRA trial (*n* = 88) [12]. The planned sample size, including a 10% drop out rate, was calculated to be 478 patients. Yet, after 3 years only 135 patients had been recruited into the study with a very limited chance of reaching the pre-calculated patient number within a reasonable period of time. This led to the decision to stop recruitment prematurely. Based on the smaller patient population the initially planned precision (lower limit of the confidence interval (CI) of the median PFS > 13.9 mos) dropped from 80 to 38.7% power. The CI width corresponding to the point estimate of 16.9 mos median PFS amounts to 8.55 mos, respectively.

Other effectiveness parameters assessed were overall survival (OS), defined as the time between diagnosis of advanced HER2+ BC and death from any cause, and overall response rate (ORR) defined as the proportion of patients with partial response (PR) or complete response (CR) as best response. Best response, defined as the best documented response during first-line palliative therapy with P was recorded as reported by the physician according to clinical routine at every infusion therapy, without response confirmation.

All variables were analyzed descriptively, displaying categorical variables with absolute and relative frequencies (%) within the respective categories. Continuous variables are reported with mean values and standard deviations (SD), including the description of 95% CI, where applicable. PFS and OS are described by Kaplan Meier method [16].

Subgroups were pre-defined for age (patients < 65 years or ≥ 65 years of age), hormone receptor status (patients with hormone receptor (HR) positive (+) and HR negative (−) BC) and metastatic status [no metastases, visceral metastases (VM), non-visceral metastases (NVM)].

A post hoc analysis of median PFS was performed by treatment-free interval (TFI) for patients with an early relapse (relapse of BC within a TFI of ≤ 6 mos) and patients relapsing after a TFI > 6 mos.

Standardized definitions for Common Terminology Criteria for Adverse Events (CTCAE) v4.03 from the National Cancer Institute (NCI) were applied for grading severity of all AEs. The Medical Dictionary for Regulatory Activities (MedDRA) v19.0/v20.0 was used for classification of reported terms within respective system organ class (SOC) and preferred terms (PT). While the eCRF was pre-populated mentioning P, this was not the case for T up until 11/2017. Thus, this should be taken into account when interpreting AE incidences for T as an actual underreporting may be assumed.

## Results

### Patient numbers and documentation

**Fig. 1:**
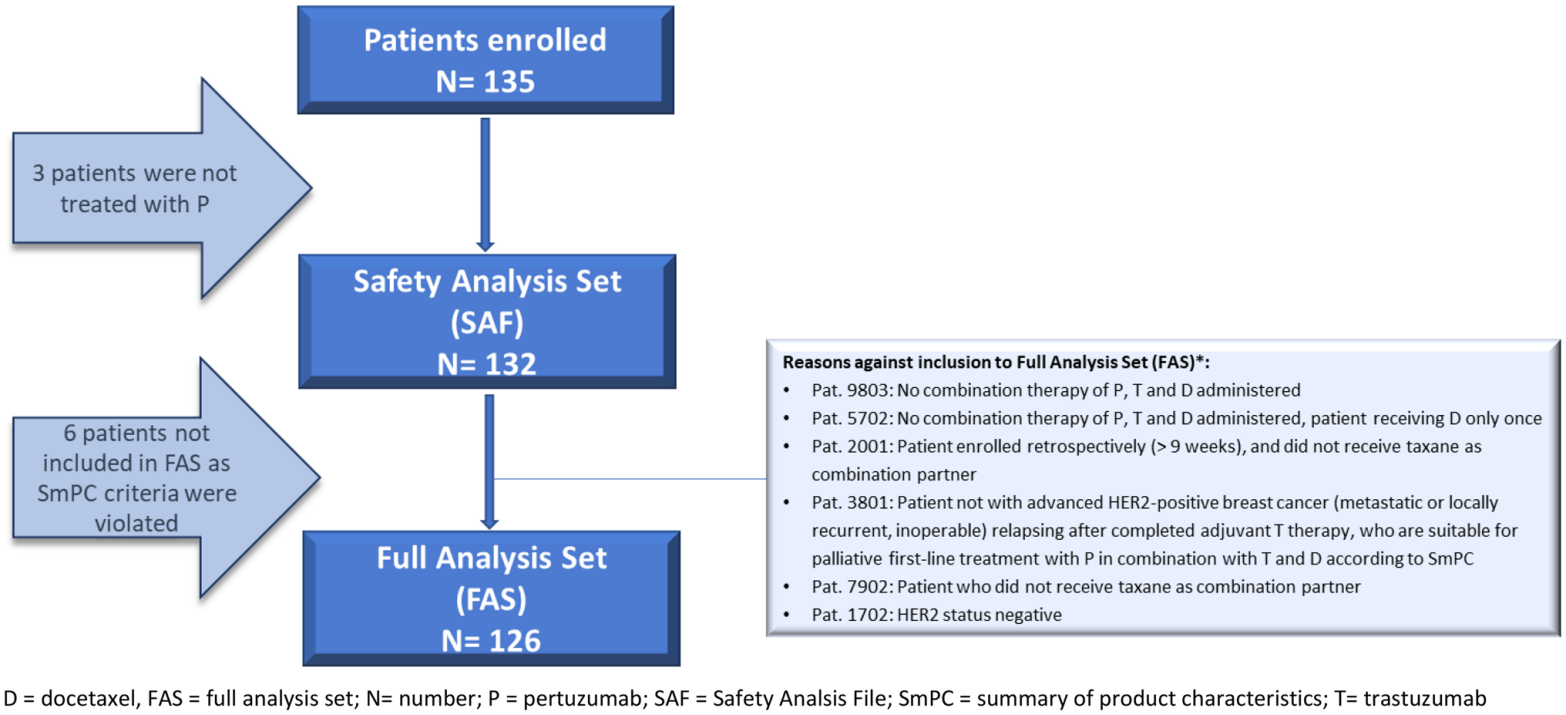
Patient disposition

Violation of inclusion criteria led to the exclusion of 21 patients out of the enregistered 156 patients (most frequent reasons were: no or not completed (neo)adjuvant T therapy (*n* = 6), unsuitable for treatment with docetaxel (*n* = 5), relapsed on adjuvant treatment with T (*n* = 2), and other reasons). Of the 135 patients enrolled, 3 were not treated with P, leaving 132 patients in the safety analysis set (SAF), comprising all eligible patients with signed ICF, who had received at least one dose of P. Another 6 patients were excluded from effectiveness assessments due to reasons displayed in Fig. 1, leaving 126 patients in the full analysis set (FAS).

**Fig. 2:**
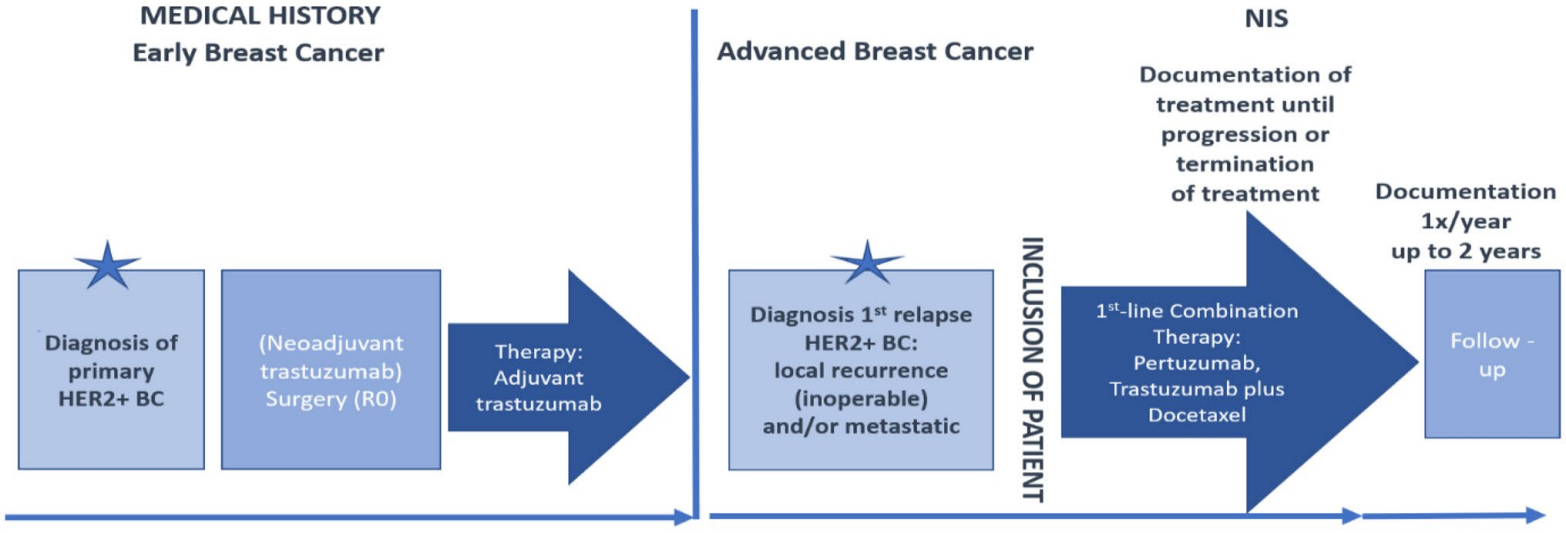
Flow chart NIS HELENA

An intense documentation period (capturing data on ECOG performance status, therapy, tumor evaluation, cardiac monitoring and safety every 3 weeks) until termination of treatment (up to a maximum of 28 cycles) or progression of disease (PD), whatever occurred first, was ensued by a follow-up documentation of up to 2 years with a once yearly assessment (Fig. 2).

The median duration of the observational period was 40.9 mos [95% CI 35.4, 43.1]. The reasons for end of intense documentation for the 126 patients were documented as follows:

- Tumor progression (*n* = 55)^a^
- Tumor remission (*n* = 2), or “no progression up to and including visit 28” (*n* = 35)^b^
- Death of patient (*n* = 11)^c^
- AE: related to therapy (*n* = 9)^d^ and AE not related to therapy (*n* = 1)
- Patient‘s wish (not toxicity related) (*n* = 5)
- Other reason (*n* = 7)^e,f^
- Missing (*n* = 1)

^a^Including 2 patients with a fatal SAE reported during study conduct elsewhere in the eCRF: (respiratory failure, and organ failure).

^b^Including 1 patient with altogether 3 fatal SAEs reported during study conduct elsewhere in the eCRF: (hepatic encephalopathy, jaundice, dyspnea).

^c^For 7 patients fatal SAEs during study conduct were reported (PTs: death, malignant neoplasm progression, embolic pneumonia, cerebral disorder, metastases to central nervous, metastasis to central nervous system).

^d^9 patients with (*n* = 71) reported drug-related events.

^e^Free text entries.

^f^Including 1 patient with a fatal SAE (death) reported during study conduct elsewhere in the eCRF.

### Patient and disease characteristics

Patient disposition, disease characteristics and prior therapies of the FAS including the pre-defined subgroups of HR ± and patients below 65 and ≥ 65 years are shown in Table 1.

**Table 1:** Patient and disease characteristics of the FAS and pre-defined subgroups

Population	Total FAS *n* = 126	Patients < 65 years *n* = 103	Patients ≥ 65 years *n* = 23	HR+^a^ *n* = 65	HR−^a^ *n* = 58
Mean age (FAS) in years (SD)	55.3 (SD 10.78)	51.8 (SD 8.34)	71.0 (SD 4.86)	54.3 (SD 10.15)	56.5 (SD 10.96)
ECOG at baseline					
0	65 (51.6%)	58 (56.3%)	7 (30.4%)	36 (55.4%)	28 (48.3%)
1	43 (34.1%)	32 (31.1%)	11 (47.8%)	21 (32.3%)	21 (36.2%)
2	7 (5.6%)	4 (3.9%)	3 (13.0%)	3 (4.6%)	4 (6.9%)
3	3 (2.4%)	2 (1.9%)	1 (4.3%)	1 (1.5%)	2 (3.4%)
Not done	8 (6.3%)	7 (6.8%)	1 (4.3%)	4 (6.2%)	3 (5.2%)
Hormone receptor status					
HR+ (eBC)	75 (59.5%)	63 (61.2%)	12 (52.2%)	65^a^ (100%)	
HR+ at NIS inclusion	41 (32.5%)	36 (35.0%)	5 (21.7%)	41 (63.1%)	
HR− (eBC)	46 (36.5%)	36 (35%)	10 (43.5%)		58^a^ (100%)
HR− at NIS inclusion	42 (33.3%)	33 (32.0%)	9 (39.1%)		42 (72.4%)
HR unknown	3 (2.9%)				
Patients with distant metastases	115 (91.3%)	93 (90.3%)	22 (95.7%)	59 (90.8%)	54 (93.1%)
Metastatic status					
Visceral metastases	92 (73.0%)	73 (70.9%)	19 (82.6%)	47 (72.3%)	44 (75.9%)
Non-visceral metastases	23 (18.3%)	20 (19.4%)	3 (13.0%)	12 (18.5%)	10 (17.2%)
No metastases	11 (8.7%)	10 (9.7%)	1 (4.3%)	6 (9.2%)	4 (6.9%)
HER2 IHC score primary tumor					
Positive+++	114 (90.5%)	96 (93.2%)	18 (78.3%)	59 (90.8%)	53 (91.4%)
Positive++^b^	2 (1.6%)	2 (1.9%)	0	1 (1.5%)	1 (1.7%)
Negative	4 (3.2%)	1 (1.0%)	3 (13.0%)	4 (6.2%)	0
Not examined/not evaluable	8 (6.4%)	4 (3.8%)	2 (8.6%)	1 (1.5%)	4 (6.8%)
Re-testing of HER2 IHC at baseline					
Positive+++	82 (65.1%)	68 (66.0%)	14 (60.9%)	40 (61.5%)	41 (70.7%)
Positive++^b^	1 (0.8%)	1 (1.0%)	0	0	1 (1.7%)
Negative	1 (0.8%)		1 (4.3%)	1 (1.5%)	
Not examined	2 (1.6%)	1 (1.0%)	1 (4.3%)	2 (3.1%)	
No re-testing	40 (31.7%)	33 (32.0%)	7 (34.7%)	22 (33.8%)	16 (27.6%)
Method of surgery of primary tumor breast conserving	56 (44.4%)	46 (44.7%)	10 (43.5%)	31 (47.7%)	25 (43.1%)
Mastectomy	51 (40.5%)	42 (40.8%)	9 (38.7%)	25 (38.5%)	24 (41.3%)
Other	9 (7.1%)	9 (8.7%)	0	7 (10.8%)	2 (3.4%)
Missing	8 (6.3%)	5 (4.9%)	3 (13.0%)	1 (1.5%)	6 (10.3%)
Resectability of primary tumor					
R0	113 (89.7%)	90 (87.4%)	23 (100%)	59 (90.8%)	51 (87.9%)
R1	1 (0.8%)	1 (1.0)	0	1 (1.5%)	0
RX	3 (2.4%)	3 (2.9%)	0	1 (1.5%)	2 (3.4%)
Unknown	9 (7.1%)	9 (8.7%)	0	4 (6.2%)	5 (8.6%)
Time (mean, years) from primary diagnosis to start of therapy with P	4.7 (SD 2.27)	4.4 (SD 2.22)	5.7 (SD 2.26)	5.0 (SD 2.32)	4.3 (SD 2.19)
Any concomitant disease	89 (70.6%)	69 (67.0%)	20 (87.0%)	44 (67.7%)	44 (75.9%)

*eBC* early breast cancer, *ECOG* eastern cooperative oncology group, *FAS* full analysis set, *HER2* human epidermal growth factor receptor 2, *IHC* immunohistochemistry, *HR* hormone receptor, *P* pertuzumab, *n* number, *NIS* non-interventional study, *SD* standard deviation, *T* trastuzumab

^a^HR status at start of NIS in case available (*n* = 83), otherwise HR status at initial diagnosis was used (*n* = 40). 13 patients had a switch HR positive to negative, and 3 patients a switch HR negative to positive.HR status in NIS unknown in 3 patients

^b^IHC (++) patients have a positive FISH or CISH test. In case IHC is negative, not evaluable/unknown or not investigated all patients have a positive FISH or CISH test, either for the primary tumor or at the re-test

Two patients were negative for HER2 by immunohistochemistry IHC (with no FISH fluorescence or chromogenic in situ hybridization or CISH test) before the start of first-line therapy with P+T, and it remains unclear, why they received adjuvant treatment with T. However, at the time of inclusion into this NIS both patients had documented HER2+ BC, one by IHC (+++) and the other one by FISH. HR−status switched from HR+ to HR− in 13 and from HR− to HR+ in 3 patients between primary diagnosis of BC and inclusion into NIS HELENA.

### Treatment—prior (neo)adjuvant therapy and disease-free interval and therapy-free interval

123 patients (FAS) had completed one year of (neo)adjuvant therapy with T. Prior neoadjuvant chemo-immunotherapy treatment of HER2+ BC was administered in 54 patients (42.9%). The most frequently used chemotherapy drugs were cyclophosphamide (*n* = 95, 75.4%), epirubicin (*n* = 77, 61.1%), docetaxel (*n* = 61, 48.4%), and paclitaxel (*n* = 41, 32.5%). Overall, 88 patients (69.8%) had an anthracycline and taxane-based chemotherapy combination. 79 (62.7%) patients received prior (neo)adjuvant endocrine therapy, most frequently tamoxifen (*n* = 53, 42.1%), followed by letrozole (*n* = 16, 12.7%) and anastrozole (*n* = 15, 11.9%). Prior adjuvant radiotherapy was applied in 95 (75.4%) patients. Median duration of prior (neo)adjuvant therapy with T was 11.8 [min. 0.0–max. 16.3] mos. The median disease-free interval (i.e., period between (R0) tumor resection before prior adjuvant T therapy and documented relapse) was 40.2 [min. 6.6–max. 95.9] mos. Median therapy-free interval (TFI), defined as last (neo)adjuvant T dose and start of therapy with P, was 29.9 [min. 1.1–max. 89.2] mos.

### Treatment patterns HER2-directed first-line therapy (FAS)

Patients (FAS) received a median of 18 [min. 1–max. 28] cycles of P and were treated with P for a median of 13.4 [95% CI 11.3, 16.3] mos. Median initial dose of P was 840 mg [min. 420–max. 844 mg], while the median first maintenance dose of P was 420 mg [min. 420–max. 840 mg], with a relative dose intensity of P of 96.2% [min. 67.0–max. 106.5%]. 71 (56.3%) patients were reported with a modification: 24 (19.0%) patients with interruption and 38 (30.2%) patients had a delay of P therapy, one patient (0.8%) was reported with a dose reduction. In 21 (16.7%) patients P therapy, and in 17 patients (13.5%) T therapy was prematurely discontinued. Table 2 provides details on all combination therapy components.

**Table 2:** Details on therapy, dosing, treatment adjustment, and treatment duration (FAS)

Details	Pertuzumab *n* = 126		Trastuzumab *n* = 126*		Docetaxel *n* = 126**	
	Median/no.	Min–max (%)	Median/no.	Min–max (%)	Median dose intensity/no.	Min–max (%)
Initial dose (median)	840 mg	420–844 mg	8.0 mg/kg	2.0–9.2 mg/kg	27.2 mg/m^2^/week	8.3–499.9 mg/m^2^/week^a^
Maintenance dose (median)	420 mg	420–840 mg	6.0 mg/kg	1.9–8.0 mg/kg		
Relative dose intensity (median)	96.2%	67.0%–106.5%	94.3%	68.8%–108.3%	27.2 mg/m^2^/week	8.3–499.9 mg/m^2^/week
Dose modification (patients)	71	56.3%	70	55.6%	79	62.7%
Reason: AE related to therapy	12	9.5%	14	11.1%	29	23.0%
Dose interruption (patients)	24	19.0%	17	13.5%	17	13.5%
Reason: AE related to therapy	6	4.8%	5	4.0%	6	4.8%
Dose reduction (patients)	1	0.8%	14	11.1%	29	23.0%
Reason: AE related to therapy	0	0	2	1.6%	16	12.7%
Therapy delay (patients)	38	30.2%	45	35.7%	13	10.3%
Reason: AE related to therapy	3	2.4%	7	5.6%	4	3.2%
Premature discontinuation of therapy (patients)	21	16.7%	17	13.5%	48	38.1%
Reason: AE related to therapy	0	0	1	0.8%	13	10.3%
Number of treatment cycles (median)	18	1–28	19	1–28	6	1–16
Duration of therapy (median, mos)	13.4	11.3–16.3	14.3	12.2–17.1	4.1	4.1–4.2

*AE* adverse event, *max* maximum, *min* minimum, *mos* months, *no.* number

*All patients had intravenous H-therapy, 3 patients received H subcutaneously, 2 of them also intravenously

**Dose intensity could not be estimated for 2 patients due to missing body height/weight. One patient was excluded from the analysis as only paclitaxel and no docetaxel therapy had been documented

^a^Dose range seems wide. As it is part of NIS documentation it might be due to a documentation error, while median dose intensity seems plausible

Efficacy (*n* = 71; 56.3%) or tolerability (*n* = 66; 52.4%) in the (neo)adjuvant treatment setting and study results and publications (*n* = 79; 62.7%), were the main reasons for choice of therapy as documented by the treating physician according to eCRF.

Overall, results from this NIS show a good therapy adherence to recommendations from the summary of product characteristics of the respective components of the HER2-directed first-line therapy.

### Safety results

Data on safety are reported for the SAF (*n* = 132). An overview of AEs, NCI CTCAE grading and related AEs, including number of fatal AEs is shown in Table 3*.*

**Table 3:** Overall safety-results and according to severity grading in SAF; patient- and case-based analysis

Total patients (SAF) *N* = 132/total events *N* = 936	Overall		CTCAE grade 1/2		CTCAE grade 3/4		CTCAE grade 5^b^	
	Patients (*n*, %)	Cases/events (*n*)	Patients (*n*, %)	Cases/events (*n*)	Patients (*n*, %)	Cases/events (*n*)	Patients (*n*, %)	Cases/events (*n*)
All AEs	**124** (93.9%)	936	**121** (91.7%)	834	**39** (29.5%)	94	**7** (5.3%)	8
ADR related to P	**71** (53.8%)	279	**69** (52.3%)	239	**19** (14.4%)	37	**2** (1.5%)	3
ADR related to T^a^	**27** (20.5%)	78	**24** (18.2%)	69	**8** (6.1%)	9	**0**	0
ADR related to D	**58** (43.9%)	228	**57** (43.2%)	202	**12** (9.1%)	26	**0**	0
All SAEs	**43** (32.6%)	87	**19** (14.4%)	26	**28** (21.2%)	53	**7** (5.3%)	8
SADR related to P	**18** (13.6%)	35	**7** (5.3%)	8	**12** (9.1%)	24	**2** (1.5%)	3
SADR related to T^a^	**9** (6.8%)	13	**6** (4.5%)	7	**5** (3.8%)	6	**0**	0
SADR related to D	**10** (7.6%)	19	**5** (3.8%)	6	**7** (5.3%)	13	**0**	0

*ADR* adverse drug reaction, *AE* adverse event, *CTCAE* common terminology criteria for adverse events, *D* docetaxel, *N/n* number, *P* pertuzumab, *SADR* serious adverse drug reaction, *SAE* serious adverse event, *SAF* safety analysis set, *T* trastuzumab

^a^As side effects to T were not pre-populated (as were for P), a certain underreporting of AE-frequency for T has to be taken into account within this NIS

^b^8 patients with a treatment-emergent fatal SAE were reported in this NIS, the CTCAE grade for one of these patients was not documented by the investigator, therefore not listed in this table

**Fig. 3:**
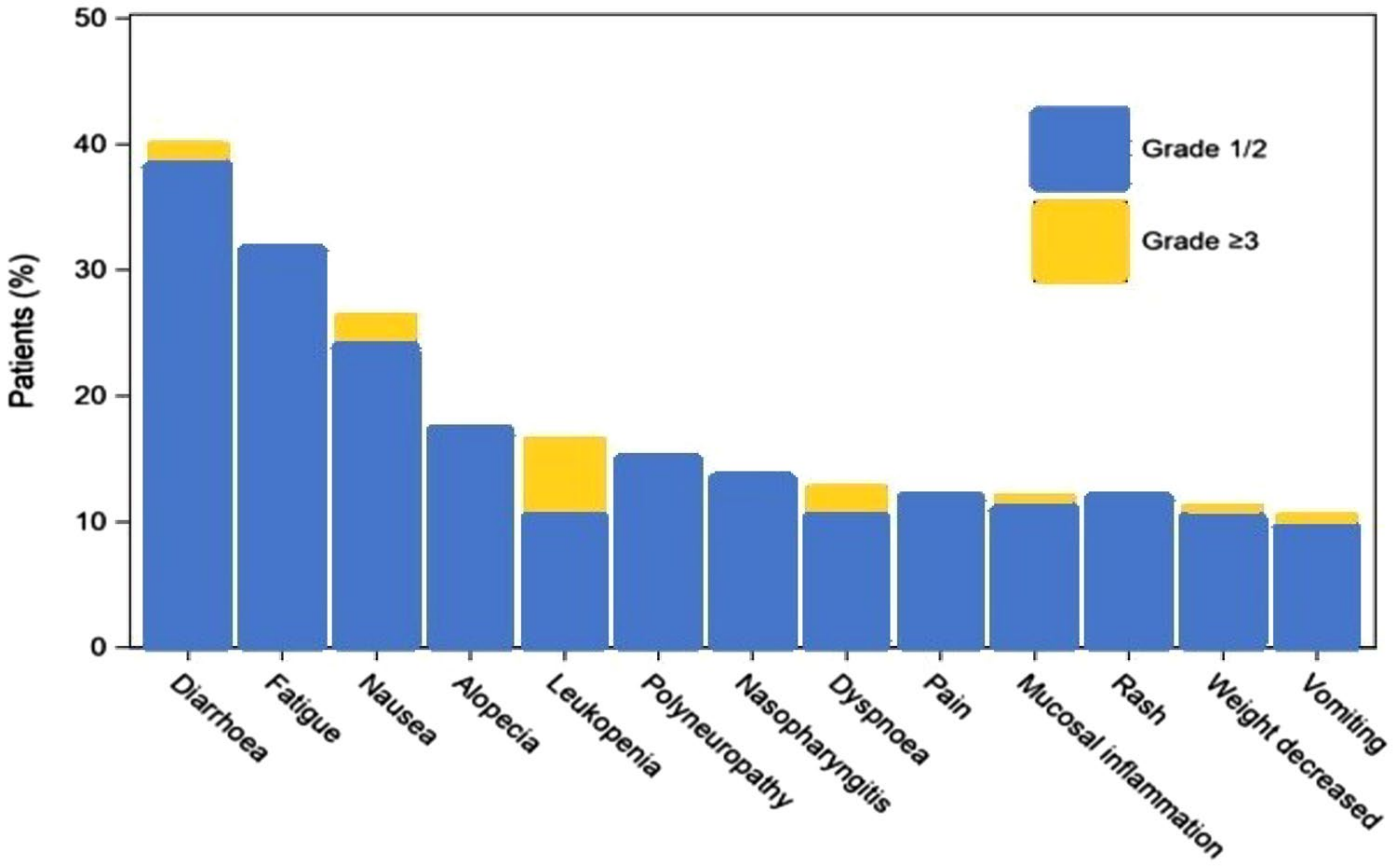
overview of most frequently reported PTs for SAF according to severity grade

In total, 8 (6.1%) patients were reported with a fatal SAE. The most frequent fatal events [documented per preferred terms (PTs)] were death and metastases to central nervous system (each *n* = 2; 1.5%). The most frequently reported any-grade PTs in SAF were diarrhea, fatigue, and nausea (Fig. 3).

**Fig. 4:**
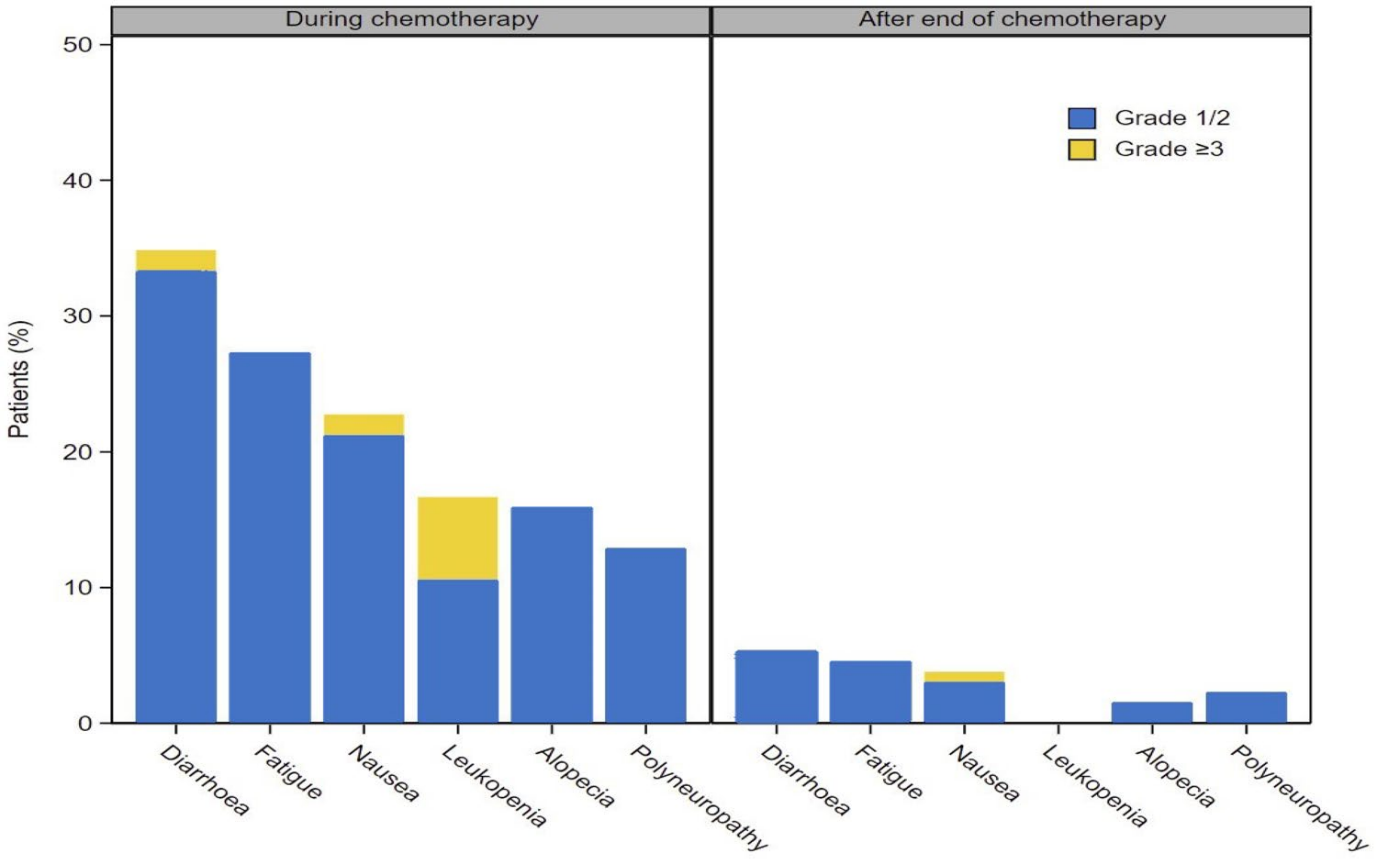
Most frequent PTs (any grade) during and after chemotherapy administration

Figure 4 displays the incidence of frequent PTs before and after finalization of chemotherapy, depicting the typical decline of chemotherapy-related AEs after discontinuation of the chemotherapy component.

### Left ventricular ejection fraction (LVEF) and cardiac function

Treatment with P and T may increase the risk for developing cardiac dysfunction. No patient was documented with a left ventricular ejection fraction (LVEF) below 50% at baseline. During the intense documentation period 11 patients (8.3%) were reported with an LVEF < 55%. LVEF was reported as pathological by the treating physician in 9 (6.8%) patients during the intense documentation period and in 13 (9.8%) patients overall (i.e., intense documentation period plus follow-up period). For 6 of these patients (with 7 events in total), this was documented as a P-related AE. All but one of the 7 reported AEs resolved.

Discontinuation of P and T therapy due to congestive heart failure was reported in 1 (0.8%) patient. Overall, cardiac events were rare.

No new safety signals, including cardiac safety were detected in clinical routine practice during this NIS.

### Effectiveness

**Fig. 5:**
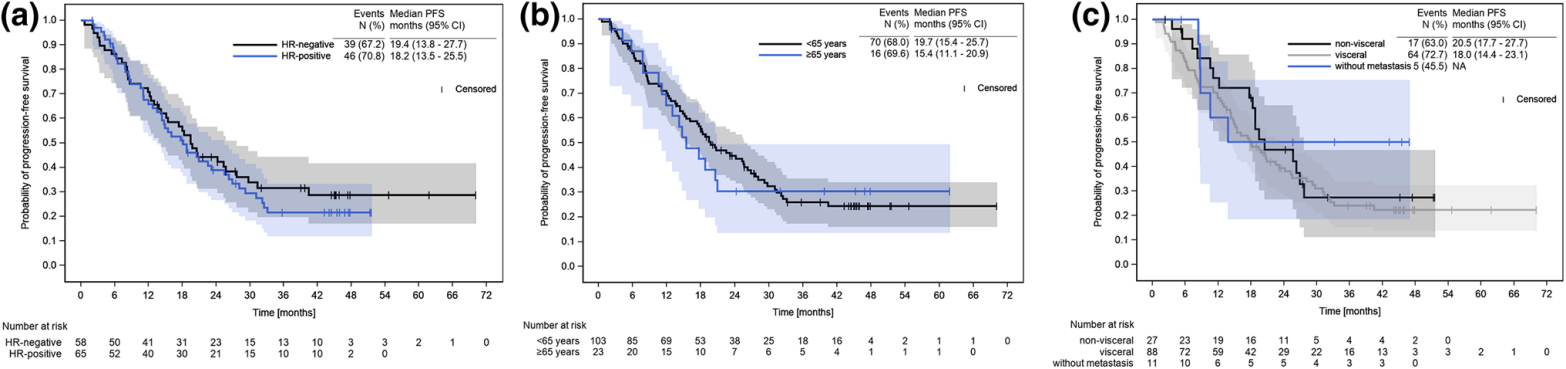
**a** PFS in HR+/HR− patients, **b** in patients < 65 and ≥ 65 years of age and **c** patients with VM, NVM and no distant metastases; with no statistical difference in the PFS between the subgroups (log-rank test; *p*_a_ = 0.522, *p*_b_ = 0.833, *p*_c_ = 0.415)

First-line dual HER2 blockade plus taxane resulted in a median PFS of 18.8 mos [95% CI 15.1, 24.2]. Median PFS in the HR+ subpopulation was 18.2 [95% CI 13.5, 25.5] mos and 19.4 [95% CI 13.8, 27.7] mos in HR− patients. Median PFS in the subgroup of patients aged < 65 years was 19.7 [95% CI 15.4, 25.7] and in patients aged ≥ 65 years 15.4 [95% CI 11.1, 20.9] mos. Similar results were obtained in the subgroup of patients with VM 18.0 [14.4, 23.1] and in patients with NVM 20.5 [17.7, 27.7] mos, while the median PFS in patients without metastases (*n* = 11) was not reached. The highest proportion of patients with a PFS event was seen in the subgroup of patients with VM (*n* = 88; 72.7%) (Fig. 5).

Overall, 46 patients died (36.5%) and 80 patients (63.5%) were censored for OS, most frequently (*n* = 65, 81.3%) at the last contact date within this NIS. A median OS for the FAS of 55.9 [41.2, not reached (n.r.)] mos was documented. Median OS for none of the pre-defined subgroups has been reached (</≥ 65 years of age, HR−/HR+, no metastases/VM/NVM) while many patients have been censored due to end of observation.

ORR results for the FAS and the pre-defined subgroups are presented in Table 4. The post hoc analysis of effectiveness parameters by TFI is provided in Table 5.

**Table 4:** Overall response rate (FAS)

Overall Response Rate (ORR), *n* = % with CR and PR			
Patient cohort	*N* =	ORR %	95%CI
Full Analysis Set (FAS)	**126**	64.3	**[55.6, 72.1]**
Hormone receptor+	65	69.2	[57.2, 79.1]
Hormone receptor−	58	60.3	[47.5, 71.9
Visceral metastases	88	68.2	[57.9, 77.0]
Non-visceral metastases	27	55.6	[37.3, 72.4]
Without metastasis	11	54.5	[28.0, 78.7]
< 65 years	103	65.0	[55.5, 73.6]
≥ 65 years	23	60.9	[40.8, 77.8]

*CI* confidence interval, *CR* complete response, *FAS* full analysis set, *N* number, *ORR* overall response rate, *PR* partial response

**Table 5:** Effectiveness parameters (FAS) according to post hoc analysis by TFI

Parameter	FAS total *n* = 126	TFI ≤ 6 mos *n* = 5	TFI > 6 mos *n* = 120	TFI unknown *n* = 1
Median PFS [95% CI] mos	18.8 [15.1, 24.2]	12.4 [3.2, NA]	19.4 [15.4, 25.5]	NA [NA, NA]
N. of events, *n*, %	86 (68.3%)	5 (100%)	81 (67.5%)	0 (0.0%)
Median OS [95% CI] mos	55.9 [41.2, NA]	34.1 [3.2, NA]	55.9 [41.2, NA]	NA [NA, NA]
N. of events, *n*, %	46 (36.5%)	2 (40.0%)	44 (36.7%)	0 (0.0%)
ORR rate (%), 95% CI	64.3% [55.6, 72.1]	60.0% [23.1, 88.2]	64.2% [55.3, 72.2]	100.0% [20.7, 100.0]

*CI* confidence interval, *FAS* full analysis set, *mos* months, *N* number, *NA* not applicable, *ORR* overall response rate, *OS* overall survival, *PFS* progression-free survival, *TFI* therapy-free interval

## Discussion

We were interested in how far the outcomes of first-line dual blockade in patients with advanced HER2+ BC and prior adjuvant T corresponded to results reported from the RCT CLEOPATRA focusing primarily on PFS. In our observational study the median PFS in the FAS was 18.8 mos, while in the CLEOPATRA trial P plus T plus D treatment of the 88 patients with prior (neo)adjuvant T resulted in a median PFS of 16.9 months [12]. Thus, with respect to PFS, the outcome of first-line dual blockade in patients with advanced HER2+ BC and prior adjuvant T from routine clinical practice match those reported in the randomized study setting.

In addition, data from our NIS provide further information on median PFS in specific patient subgroups defined by age or certain disease-related characteristics. Yet, our data do not allow a statistical distinction of subgroups benefitting to a greater or lesser extent from first-line dual HER2 blockade. A supposedly better effectiveness could have resulted from an enhanced treatment response or may equally be based on the underlying better prognosis of a certain patient subgroup.

In our study HR− and HR+ patients showed similar median PFS results (HR− 19.4 [95% CI 13.8, 27.7], HR+  18.2 [95% CI 13.5, 25.5] mos). This was somewhat unexpected as 10% of HR− patients had an ECOG performance status of 2 or 3, while only 6.1% of HR+ patients presented with an ECOG > 1 at inclusion. On the other hand, a markedly higher proportion of HR– patients had undergone a mastectomy (24.1%) as compared to the subgroup of HR+ patients (12.3%).

PFS results were comparable in patients without metastases (*n* = 11), in patients with NVM (*n* = 23) and those with VM (*n* = 92) (no metastases: NR; NVM: 20.5 [17.7, 27.7]; VM: 18.0 [14.4, 23.1] mos), though patients with mBC and VM tend to have a worse survival prognosis [17, 18].

With a median PFS of 19.7 [95% CI 15.4, 25.7] mos, younger patients (< 65 years of age) had slightly better PFS outcomes than patients aged ≥ 65 years with a median PFS of 15.4 [95% CI 11.1, 20.9] mos. This may be linked to the well-known multimorbidity and higher frailty in older patients and was endorsed by a higher percentage of patients with an ECOG ≥ 1 in the subgroup of patients aged ≥ 65 years in this NIS.

In our post hoc analysis we observed a median PFS of only 12.4 mos for patients with an early relapse, defined by TFI ≤ 6 mos, clearly supporting the prognostic relevance of a short TFI [6].

The ORR (64.3%) obtained in our NIS was lower as compared to the ORR (80.2%) reported for the CLEOPATRA study [19]. However, due to the observational setting NIS HELENA did not mandate to assess response per pre-defined criteria such as RECIST or at defined regular intervals. ORR in the subgroups of HR+ , HR−, VM or, NVM patients and patients aged < 65 years or ≥ 65 years seems to be comparable given the marked overlap of the 95% CIs.

The estimated median observational period in this NIS is 40.9 mos. Eighty (63.5%) patients were still alive at the end of the observation period. Therefore, the calculated median OS of 55.9 [41.2,n.r.] mos relates to a high number of censored patients. It is in line with the median OS of 56.5 mos reported for the overall patient population of the CLEOPATRA study after 50 mos of follow-up [19]. Yet, the reported result from CLEOPATRA comprises outcomes from patients with and without prior (neo)adjuvant T. Plus, patient and disease characteristics were more formally standardized based on defined in- and exclusion criteria.

QoL results from our NIS are not displayed here and have to be interpreted with caution, due to the observational setting and the declining response rates through the course of the study. However, an initial worsening of the values at week 24 with a subsequent recovery to baseline values and above corresponds with the observed decline of chemotherapy-related AEs after discontinuation of the chemotherapy component.

Safety results from this NIS principally reflect those reported from the clinical trials on first-line dual HER2 blockade with P plus T plus chemotherapy. However, due to the design of documentation a certain underreporting, especially regarding T-related side effects cannot be ruled out. Plus, the observational character of the study per se may favor underreporting of adverse events. Overall, the safety profile, including cardiac safety, from our NIS corresponds with the safety data reported from clinical trials like CLEOPATRA [19] or PERUSE [20]. It is also consistent with the known safety profiles of the single drugs. Thus, no new safety signals were detected during the conduct of this NIS. The known safety profiles of the HER2-directed antibodies were confirmed. In particular, cardiac safety was comparable even though patients were re-treated with HER2-directed antibodies.

Our NIS has a number of limitations. There are limitations in the formal study set-up such as the non-interventional setting and the lack of randomization, of source data validation, of standardized assessments of treatment outcomes, or of a structured visit and follow-up planning. Parameters pre-populated in the eCRF, as it was handled only for P initially, will receive more attention than those that need to be entered manually. Our NIS did not reach the pre-planned patient number due to slow recruitment. This may have been due to the requirements to follow the respective specifications of the summary of product characteristics and to select patients with completed (neo)adjuvant pre-treatment. Access to prior (neo)adjuvant HER2-directed therapy probably reduced the number of patients with an early relapse of HER2+ BC in clinical practice. This can explain the comparatively low number of patients with an early relapse documented in our NIS. Patients selected for this NIS were principally at a high risk of disease progression. The number of patients actually recruited into our NIS allows to differentiate between patient subgroups descriptively to some extent, yet without any statistical significance. Differences in outcomes may be a treatment effect but can also relate to one or more patient or disease characteristics with prognostic impact. Finally, the duration of the observation period precluded a robust OS projection.

## Conclusions

The patients from NIS HELENA were treated with first-line dual HER2 blockade after having received HER2-directed treatment with T in the (neo)adjuvant setting. Effectiveness and safety of re-treatment with T had been reported from clinical trials and from observational studies previously [21–23]. Outcomes of re-treatment with HER2-antibodies are now available for first-line dual blockade after prior (neo)adjuvant HER2-antibody therapy from clinical routine practice. Data for the pre-defined patient subgroups may further elucidate effectiveness of the dual blockade in patients with clinically relevant disease characteristics.

## Data Availability

For up to date details on Roche’s Global Policy on the Sharing of Clinical Information and how to request access to related clinical study documents, see here: https://go.roche.com/data_sharing
